# “Doing My Part in My Healing”: A Qualitative Study Exploring Integrative Oncology Practices Among African Americans with Cancer

**DOI:** 10.1177/27536130261468343

**Published:** 2026-07-22

**Authors:** Chanda L Williams, Adrienne Yang, Serena Gilmore, Dalila Stanfield, Paige Steiding, Chloe E. Atreya, Sorbarikor Piawah

**Affiliations:** 1 673835 UCSF Osher Center for Integrative Health, San Francisco, US; 2 UCSF School of Medicine, San Francisco, US; 3 UCSF Helen Diller Comprehensive Cancer Institute, San Francisco, US

**Keywords:** integrative medicine, integrative oncology, African Americans, health disparities, integrative health equity, cancer disparities

## Abstract

**Background:**

African Americans in the United States continue to experience disproportionate cancer mortality and lower survival across cancer types. Integrative oncology (IO) may help address symptom burden, treatment-related toxicities, and quality of life. However, African Americans remain underrepresented in IO research and may face barriers to access.

**Objectives:**

To explore experiences with and attitudes toward IO among African American adults with cancer and to assess interest in participating in IO research.

**Methods:**

We conducted a qualitative cross-sectional descriptive study with African American adults receiving cancer care in the San Francisco Bay Area. Data sources included 17 individual semi-structured interviews, one patient focus group (n=5), and one Community Advisory Board focus group (n=3). The patient focus group participants had previously completed interviews. Study participants had a median age of 53 years (range 32–66), 71% were women, and represented a range of cancer diagnoses, treatment experiences, educational backgrounds, and employment statuses. Data were analyzed using a grounded theory–informed thematic approach, with interviews and focus groups coded by source and integrated during thematic development.

**Results:**

Four themes were identified: (1) participants described cancer care within the context of interpersonal and structural racism; (2) participants used a broad range of integrative and complementary approaches; (3) despite high interest in IO, access was limited by lack of awareness, scheduling constraints, and cost; and (4) participants expressed strong interest in IO research, while emphasizing the importance of trust, transparency, and community benefit.

**Conclusion:**

African American adults with cancer expressed a strong interest in IO and IO research but described substantial barriers to access and participation. These findings underscore the need for more accessible, trustworthy, and culturally responsive approaches to IO delivery and research.

## Introduction

Integrative oncology (IO) is a patient-centered, evidence-based approach that incorporates mind-body practices, lifestyle interventions, and natural products alongside conventional cancer treatment to improve symptom management, quality of life, and overall outcomes.^1-9^ Recent joint guidelines from the Society of Integrative Oncology and the American Society of Clinical Oncology underscore the growing role of IO in comprehensive cancer care.^1,2,10-12^ As patients increasingly engage in culturally relevant and cost-effective integrative therapies, there is a critical need to ensure that IO delivery is equitable and responsive to diverse populations.

Cancer remains a significant public health burden in the United States, with over 2 million new cases and more than 600,000 deaths projected annually.^13^ African Americans experience a disproportionate burden of cancer, including higher mortality rates, worse survival outcomes, and greater symptom burden compared to White individuals.^10,14-25^ These disparities are driven by multilevel factors, including comorbid conditions, inequities in access to high-quality screening and treatment, and the enduring effects of structural racism on socioeconomic conditions and healthcare access.^19,26-30^ African American patients also report worse cancer-related quality of life, including greater pain severity, fatigue, and reduced physical functioning.^31-36^

Despite the potential for IO to address symptom burden and improve quality of life, inequities in access, utilization, and research participation persist.^37,38^ African American patients remain underrepresented in IO research, limiting the generalizability of findings and the development of culturally responsive interventions.^37,39^ Given the alignment of many IO modalities with culturally grounded healing practices, expanding equitable access to IO may represent a critical pathway for addressing disparities in cancer outcomes and survivorship.

To address these gaps, we conducted a cross-sectional qualitative descriptive study among African American individuals with cancer. The National Center for Complementary and Integrative Health (NCCIH) currently classifies complementary health approaches into four broad categories: nutritional, psychological, physical, and combination approaches.^40^ This framework reflects an evolution from earlier terminology that distinguished between natural products and mind-body practices. In this manuscript, we use NCCIH’s current classification when describing complementary and integrative health approaches, while retaining historical terminology when discussing prior studies and literature. The research is presented according to the NCCIH classification of the five CAM modalities: mind-body (e.g., biofeedback, meditation, yoga), body-based (e.g., chiropractic, reflexology), alternative medical systems (e.g., Chinese Traditional Medicine, homeopathy), energy therapies (e.g., Reiki, breathing techniques), and natural products (e.g., herbal and vitamin supplements).^41-43^ The primary objective was to examine the uptake and acceptability of IO practices and complementary therapies. Secondarily, we explored attitudes toward IO research to assess the feasibility of recruiting African American patients into IO intervention studies. Findings from this study will inform the design of future interventions to advance integrative health equity in oncology.

## Methods

### Study Design and Setting

From 2021 through 2023, we conducted a cross-sectional qualitative descriptive study to examine experiences with and attitudes toward integrative oncology (IO) among African American adults with cancer. The study was conducted in the San Francisco Bay Area and included participants receiving care at Zuckerberg San Francisco General Hospital (ZSFG) and the University of California, San Francisco (UCSF), as well as members of the Helen Diller Family Comprehensive Cancer Center (HDFCCC) Community Advisory Board (CAB). The UCSF Institutional Review Board approved all study procedures.

The study included three complementary data sources: individual semi-structured interviews, a patient focus group, and a CAB focus group. Individual interviews elicited in-depth personal experiences with cancer care, IO use, barriers to access, and attitudes toward research participation. The patient focus group deepened F of emerging themes and intervention preferences. The CAB focus group contributed broader community and implementation perspectives, including recruitment strategies, community trust, dissemination, and the acceptability of IO interventions.

### Positionality and Reflexivity

The study was led by an African American principal investigator with expertise in integrative health and qualitative research. The research team included African Americans and racially diverse members involved in recruitment, interviewing, and analysis. Racial concordance was considered important for fostering trust and facilitating open dialogue on sensitive topics, including experiences of racism in healthcare.

The research team engaged in ongoing reflexive discussion throughout data collection and analysis to examine how team members’ identities, assumptions, and prior experiences may have shaped interactions with participants and the interpretation of the data.

### Sample and Recruitment

Eligible participants were adults aged 18 years or older who self-identified as Black or African American, had been diagnosed with a solid malignancy within the previous five years, were able to complete study procedures in English, and were willing to provide informed consent. The focus on individuals with solid malignancies reflected the clinical populations available at participating recruitment sites and the study’s emphasis on commonly treated cancers, where disparities in outcomes and access to supportive care are well documented. This criterion should be considered when interpreting the transferability of findings to individuals with hematologic malignancies, whose treatment trajectories and supportive care needs may differ.

CAB participants were eligible if they self-identified as Black or African American, were aged 18 years or older, and were willing to participate in a focus group.

Participants were identified through multiple recruitment strategies, including review of oncology clinic schedules, clinician referrals, recruitment flyers, and invitation letters distributed by providers. Recruitment materials included photographs of the principal investigator and the first author to foster rapport and trust. Research staff reviewed electronic health records to confirm eligibility and attempted telephone contact up to 3 times. Interested participants provided informed consent either in person or electronically. Participants who completed an interview were invited to participate in a patient focus group if interested.

### Data Collection

1. Individual Interviews

Semi-structured interviews were conducted with African American adults with cancer who met eligibility criteria. The interview guide was informed by prior literature on complementary and integrative health use among women of color, including work by Kronenberg et al, as well as by domains included in national surveys of complementary medicine use.^44,45^ These domains were adapted for qualitative inquiry and included prior and current use of integrative practices, perceived benefits and barriers, awareness of IO services, and attitudes toward participation in research.

Interviews were conducted via secure video conferencing or telephone and lasted approximately 60–90 minutes. All interviews were audio recorded, professionally transcribed, and de-identified. Participants received a $40 gift card for participation.

2. Focus Groups

Two focus groups were conducted: one with patient participants who met the eligibility criteria above and one with CAB members. Focus group guides were developed to complement the semi-structured interview and to elicit shared perspectives on IO, barriers to access, research participation, and preferences for intervention design. The focus group questions were informed by study objectives, existing integrative oncology literature, and preliminary insights from early interviews. CAB members also contributed perspectives on community engagement and recruitment strategies. Participants received a $40 gift card for participation.

Five participants in the patient focus group had previously completed individual interviews. These participants were included to deepen the exploration of emerging themes and to generate discussion about intervention preferences and research participation. Focus groups were conducted via secure video conferencing and audio recorded and transcribed.

#### Image-Sorting Exercise

**Figure 1.**
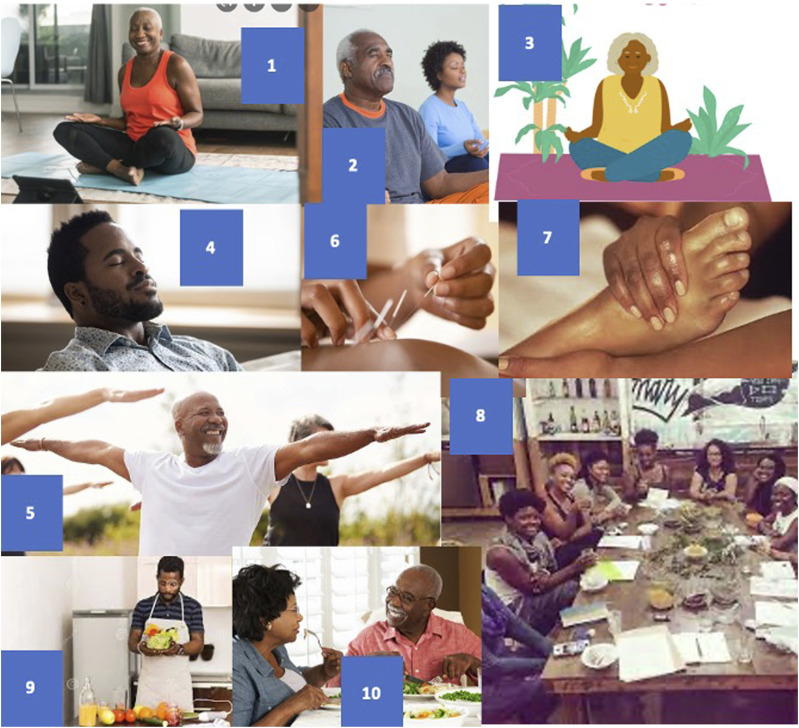
Focus Group Image Sort Image-sorting exercise conducted during the patient focus group to explore participants’ perceptions of integrative oncology. Participants selected images that represented what integrative oncology meant to them and described the meanings and emotions associated with their selections. The exercise facilitated discussion of participants’ lived experiences and conceptualizations of integrative and complementary approaches during cancer care

During the patient focus group, an image-sorting exercise was used to facilitate discussion (Figure 1). The image-sorting exercise was a visual elicitation technique used to facilitate reflection and discussion of experiential and affective dimensions of integrative oncology.^46-49^ Participants were presented with a curated set of images related to health, well-being, and integrative practices, and asked to select those that reflected their understanding of integrative oncology. Participants then described their selections and associated meanings. This technique was used to support reflection and elicit experiential and affective dimensions of IO.

### Data Analysis

Data were analyzed using an inductive thematic analysis approach informed by qualitative descriptive methods.^50-54^ Analysis proceeded iteratively and included multiple stages of coding and theme development. First, members of the research team independently reviewed transcripts to become familiar with the data and identify preliminary concepts. Second, the team developed an initial codebook through discussion of early transcripts, with attention to participants’ descriptions of cancer care, use of integrative oncology, barriers to access, racism, spirituality, trust, and research participation. Third, interviews were coded using the refined codebook, and codes were compared across interviews and focus groups to identify areas of convergence, divergence, and elaboration.

Themes were developed across the full dataset while preserving distinctions in the contributions of individual interviews, the patient focus group, and the CAB focus group. Focus group data were used to contextualize and deepen themes identified in the interviews rather than to serve as independent confirmation of their prevalence. Multiple coders participated in the analytic process, and coding discrepancies were resolved through discussion. Reflexive memos and an audit trail were maintained throughout the analysis. Data collection continued until thematic saturation was reached, defined as the point at which no new themes emerged. Descriptive statistics were used to summarize participant characteristics and baseline use of integrative practices. Reporting of the study findings adheres to the SRQR checklist; see Supplemental Material.

## Results

### Participant Characteristics

Twenty-seven individuals consented to participate. Of these, 23 completed baseline questionnaires, and 17 completed individual semi-structured interviews. The individuals who had consented to participate in the study but did not complete interviews did so due to scheduling challenges, were lost to follow-up, or had changing availability during cancer treatment and survivorship.

All interview participants were subsequently invited to a patient focus group to further explore emerging themes, and five individuals accepted. The Community Advisory Board (CAB) focus group included three members.

Seventeen African American adults with cancer participated in interviews. Participants had a median age of 53 years (range 32–66), 71% were women, and represented diverse cancer diagnoses and socioeconomic backgrounds (Table 1). Use of integrative oncology approaches was common following cancer diagnosis, with participants most frequently reporting vitamins and supplements (82%), meditation or relaxation practices (53%), herbal remedies (47%), dietary changes (41%), and movement-based therapies (41%).

**Table 1.** Demographics

Demographic characteristics	N (%)
Total Participants	17
**Age, Median (Range)**	53 (32-66)
**Gender**	
Male	5 (29%)
Female	12 (71%)
**Clinic**	
ZSFG	5 (29%)
UCSF	12 (71%)
**Cancer Diagnosis**	
GI	7 (41%)
GU	5 (29%)
Brain and Nervous System	1 (6%)
Breast	2 (12%)
Head and Neck	1 (6%)
Other Endorine	1 (6%)
**Years Since Diagnosis**	
<1 year	6 (35%)
1-5 years	10 (59%)
>5 years	1 (6%)
**Treatment Received by Time of Enrollment**	
Chemotherapy, immunotherapy, or other systemic therapy only	0 (0%)
Surgery Only	3 (18%)
Radiation Only	0 (0%)
Combination of chemotherapy or immunotherapy, surgery, and/or radiation therapy	14 (82%)
**Treatment Status at Enrollment**	
Receiving Treatment	7 (41%)
Not Receiving Treatment	10 (59%)
**Employment Status**	
Currently Working	5 (29%)
Full-time	3 (18%)
Part-Time	2 (12%)
Not Working	12 (71%)
On Disability	5 (29%)
Retired	3 (18%)
Unable to Work (Not Disabled)	2 (12%)
Temporary Leave	2 (12%)
**Highest Level of Education**	
High School or Less	9 (53%)
Bachelor’s Degree	2 (12%)
Graduate or Professional Degree	4 (24%)
Other	1 (6%)
Unknown	1 (6%)
**Marital Status**	
Single*	9 (53%)
Married or Partnered	8 (47%)

*Includes legally separated/divorced, widowed, and single.

Participants were recruited from UCSF (71%) and Zuckerberg San Francisco General Hospital (29%). Cancer diagnoses included gastrointestinal (41%), genitourinary (29%), breast (12%), brain and nervous system (6%), head and neck (6%), and other endocrine cancers (6%). Most participants (94%) had been diagnosed within the previous five years, including 35% diagnosed within the prior year. At the time of enrollment, 41% were actively receiving cancer treatment and 59% had completed treatment. The majority (82%) had undergone multimodal treatment involving chemotherapy, immunotherapy, surgery, and/or radiation.

Participants also represented diverse socioeconomic backgrounds. More than half (53%) reported a high school education or less, while 24% held graduate or professional degrees. Most participants were not currently working (71%), including individuals on disability (29%), retired (18%), temporarily on leave (12%), or unable to work (12%). Approximately half were single, divorced, widowed, or legally separated (53%), while 47% were married or partnered.

### Participation in Integrative Oncology

Participants reported engaging in a wide range of integrative oncology (IO) approaches both prior to and following their cancer diagnosis (Table 2). Use of integrative approaches increased after diagnosis, suggesting that many participants sought additional supportive care during treatment and survivorship.

**Table 2.** Use of Integrative Medicine

Approach in past 12 months	Y (N, %)		No (N, %)
	Pre-diagnosis	Post-diagnosis	
Vitamins/Supplements	6 (35%)	14 (82%)	2 (12%)
Special Diet/dietary changes	0	7 (41%)	0
Herbal Remedies	3 (18%)	8 (47%)	3 (18%)
Chinese Medicine	0	2 (12%)	0
Ayurveda	0	0	0
Indigenous Healing	0	0	0
Curanderismo	0	0	0
Homeopathy	0	0	0
Movement Therapy	2 (12%)	7 (41%)	5 (29%)
Meditation/Guided imagery/Progressive Relaxation	4 (12%)	9 (53%)	3 (18%)
Chiropractic Treatments	2 (12%)	0	5 (29%)
Massage/Acupuncture	5 (29%)	7 (14%)	3 (18%)
Energy Medicine/Reiki/Therapeutic touch	1 (6%)	2 (12%)	1 (6%)
Religion/Spirituality	13 (76%)	0	0
Other (list type by N)	Therapy, 1 (6%)	Physcial Therapist, 2 (12%)	Support Systems, 1 (6%)
	Support Systems, 9 (53%)	Podiatrist, 1 (6%)	Psychotherapy, 2 (12%)
	​	Music Therapy, 3 (18%)	Support Groups, 3 (18%)
	​	Support systems, 2 (12%)	​
	​	Therapy, 7 (41%)	​

The most reported approaches following diagnosis included vitamins and supplements (82%), meditation, guided imagery, or progressive relaxation (53%), herbal remedies (47%), special diet or dietary changes (41%), and movement-based therapies such as yoga or exercise (41%). Participants also reported using massage or acupuncture (41%), reflecting interest in both mind–body and symptom-focused interventions.

Spirituality and religion were widely used prior to diagnosis (76%), highlighting the importance of faith-based coping strategies among participants. Although spirituality was not specifically captured as a post-diagnosis category in the table, qualitative findings indicated that spirituality remained central to participants’ coping and meaning-making throughout their cancer journeys.

Some integrative approaches were used less frequently, including Chinese medicine (12%), energy medicine or Reiki (12%), and chiropractic treatments (12%). No participants reported using Ayurveda, Indigenous healing practices, curanderismo, or homeopathy within the past 12 months.

Participants also reported engaging in additional supportive services categorized as “other,” including therapy, support systems, music therapy, and physical therapy. After diagnosis, therapy (41%), music therapy (18%), and physical therapy (12%) were commonly reported, further highlighting the importance of psychosocial and supportive care during cancer treatment.

Overall, these findings demonstrate that participants engaged in diverse integrative approaches, with increased utilization following diagnosis and a preference for modalities addressing emotional well-being, symptom management, and supportive care needs.

### Overview of Themes

Four major themes were identified across interviews and focus groups:

1. Interpersonal and structural racism in cancer care
2. Integrative practices, faith, and agency in healing
3. Limited access to integrative oncology care
4. Research interest shaped by trust, transparency, and reciprocity

## Theme 1: Interpersonal and Structural Racism in Cancer Care

Participants described their cancer care experiences that occurred within the broader context of racial inequities in healthcare. While racism in cancer care has been well documented in the literature,^27,28,30,55^ participants shared personal experiences that reflected both interpersonal and systemic manifestations of these inequities, including delayed communication, inadequate symptom management, and concerns that racial stereotypes influenced clinical decision-making.

Several participants described interactions with healthcare providers that left them feeling stereotyped, dismissed, or judged. Following her diagnosis, Participant 012 recalled a clinician attributing her cancer to sexual behavior, an explanation she experienced as both inappropriate and stigmatizing: “He says… it probably was rough sex. I was speechless… I’m at a vulnerable place.” (Participant 012, Interview).

Participants also described experiences in which they felt inadequately cared for during treatment. Participant 008 recounted learning of her cancer diagnosis only after a temporary office employee contacted her because no one had informed her of the pathology findings. She later described difficulties obtaining adequate pain management following surgery:

“They wouldn’t give me pain medications because I guess they think that African American women are… I kept telling them, I just had surgery here, I didn’t come in off the streets, and I’m hurting. And for four freaking days, they would not give me anything more than like Tylenol 1000.” (Participant 008)

This participant went on to describe the experience as “the most insulting, humiliating, painful” part of her cancer journey and noted that attempts to advocate for herself were constrained by concerns about being perceived through racialized stereotypes, sharing that “sometimes you get so mad you just want to say something… because then you just look like the angry Black woman.”

Participants also recognized broader structural forms of racism within healthcare. Several participants referenced longstanding misconceptions about Black patients’ pain tolerance that they believed continued to influence clinical care. Collectively, these narratives highlighted how racism was experienced both through individual interactions with healthcare providers and through broader systemic inequities that shaped participants’ cancer care experiences. These experiences contributed to distress, mistrust, and a sense of vulnerability during an already destabilizing time.

## Theme 2: Integrative Practices, Faith, and Agency in Healing

Participants described using a broad range of integrative and complementary approaches, including acupuncture, supplements, movement-based practices, meditation, psychotherapy, music therapy, and support groups. Across interviews and focus groups, these practices were not described solely as symptom-management strategies; they were also framed as ways of reclaiming agency during cancer treatment.

As one participant explained, “I am grateful that this [IO] is available and that I have insurance that I can take advantage of it. But at the same time, I wanna feel like I am *doing my part in my healing* (007).” This statement reflects how IO was perceived as supportive care and as a means of fostering personal agency and empowerment during treatment. Several participants similarly described integrative approaches as helping them take an active role in maintaining their health and well-being:

“It makes me feel like I’m taking an active part in keeping myself healthy.” (Participant 005, Focus Group).

These perspectives highlight how integrative modalities contributed to a sense of control and participation during a time when many aspects of treatment felt uncertain or externally directed. Spirituality and faith were central to participants’ coping, meaning-making, and resilience. Participants frequently described prayer, faith, and spiritual grounding as foundational to their navigation of diagnosis, treatment, uncertainty, and survivorship. One participant stated, “I need to walk in my faith… everything I do is for Him.” (Participant 007, Interview).

For many participants, faith was not separate from healing; rather, it shaped how they understood their bodies, relationships, medical treatment, and capacity to endure. This emphasis on spirituality underscores the importance of culturally relevant coping strategies that align with participants’ values and lived experiences. Several participants had prior exposure to integrative modalities before their cancer diagnosis, while others sought them out during treatment:

“The side effect is you get the best nap while you’re getting it (acupuncture) that you’ve ever had in your life. And then when you’re done, you get a little rush, and then a couple days later, your muscles just feel good.” Participant 011, Interview).

This description highlights how integrative therapies were experienced as both restorative and therapeutic, supporting physical comfort and emotional relief during cancer treatment. Focus group discussions further emphasized the importance of connection, peace, and relational support in participants’ conceptualizations of integrative oncology. An image-sort exercise during the focus group elicited additional themes, including the importance of spirituality and relationships in their cancer journeys (Figure 1).

“Yeah, there’s three (images) that stood out to me. The one of the older woman. It looks like she’s doing yoga. That is what I was doing during my treatment.” (Participant 015), Patient Focus Group).

This reflection illustrates how participants integrated mind–body practices, such as yoga, into their cancer journeys, viewing these approaches as supportive of both physical and emotional healing. Participants expressed interest in a range of integrative approaches, particularly movement-based therapies, meditation, acupuncture, and spirituality-based practices. Participants also emphasized the importance of interventions that incorporate social support, education, and opportunities for shared experiences with other individuals undergoing cancer treatment.

## Theme 3: Limited Access to Integrative Oncology

Despite a strong interest in IO, participants described multiple barriers to access, including a lack of awareness, scheduling constraints, and cost. These structural and informational challenges often limited participants’ ability to engage with integrative approaches, even when interest and perceived benefit were high.

### Lack of Awareness

Many participants reported limited knowledge of available IO services and described a lack of guidance from clinical teams. One participant described feeling that integrative approaches were not actively supported by providers and required independent pursuit:

“The biggest challenge to access is that you are overwhelmed in the beginning [of the diagnosis], and you're grasping for answers, and you want someone to give you hope and lead you to that path of healing. They (the medical team) were kind of dismissive about it [IO]. I didn't want to have anything that was going to threaten or slow down or get in the way of what I thought was the thing that was really going to heal me. So I think that's it, not having a medical team that really understands the value of it, and appreciates it, and truly makes it a part of the treatment process. For me, it was a whole separate thing that I had to pursue on my own.” (Participant 018, Interview)

This experience suggests that the absence of provider engagement or referrals may contribute to underutilization of integrative oncology services. Participants emphasized that both patients and providers may lack awareness of IO options, which created additional barriers to access and integration into care, contributing to underutilization.

### Scheduling Constraints

Participants reported difficulty integrating IO into existing treatment schedules and long wait times for services. One focus group participant expressed disappointment at losing access to services that had been helpful:

“They only had it now and then. It (yoga) wasn’t like all the time… I really miss them.” (Participant 019, Focus Group).

This reflection highlights how the inconsistent availability of services limited sustained engagement with integrative care. Another participant described extended wait times that delayed access to integrative therapies:

“It took me months… I was on the waitlist for six months.” (Participant 007, Interview).

These logistical barriers suggest that even when services are available, limited capacity and scheduling challenges may prevent timely access, particularly during critical phases of treatment. Participants also expressed a preference for flexible delivery formats, including hybrid models that combine in-person and virtual participation. These preferences reflected the need to accommodate treatment schedules, transportation challenges, and fluctuating energy levels during cancer care.

### Cost

Financial barriers were frequently cited, particularly when services were not covered by insurance. One participant described insurance limitations as a barrier to accessing integrative therapies. When participants were asked if they had tried acupuncture or seen a chiropractor, they responded:

“I did. (But) because of the type of insurance I have, it’s an HMO. They don’t take it. [Insurance] doesn’t cover the acupuncture.” (Participant 016, Interview).

This statement reflects how insurance coverage and affordability shaped participants’ ability to initiate and sustain engagement with integrative oncology services. Participants described cost as limiting both initial access and continued participation, highlighting structural inequities in access to supportive care.

## Theme 4: Research Interest Shaped by Trust, Transparency, and Reciprocity

Participants expressed strong interest in participating in research, often motivated by a desire to contribute to improved care and greater representation for African Americans with cancer. One participant described their motivation in terms of service and contributing to future patients:

“I want to be of service.” (Participant 007, Interview).

This statement reflects a broader sense of altruism and community responsibility expressed by participants, who viewed research participation as an opportunity to improve care and address disparities affecting their communities. However, this interest was accompanied by concerns about trust, transparency, and the perceived extractive nature of research. Participants described past experiences in which they contributed to research but did not receive follow-up or see outcomes. One community advisory board participant expressed this concern directly:

“Don’t just talk to us, get your degree, and disappear.” (Participant 22, CAB Focus Group).

This statement highlights participants’ concerns about transactional research relationships and underscores the importance of accountability, reciprocity, and sustained engagement with communities. Participants emphasized that trust in research is strengthened when investigators maintain communication, share results, and demonstrate how findings benefit the community.

Participants also emphasized the importance of clear communication about the study’s purpose, anticipated benefits, and plans for dissemination. Trusted relationships, including clinician recommendations and community engagement, were identified as important facilitators of participation. These findings suggest that relational trust and transparency are central to improving recruitment and engagement among African American participants.

“I’m all in. That’s the least I can do is maybe help make it better for, you know, heaven forbid the next patient who has to go through this.” (Participant 011, Interview).

“I’m often not familiar with what [researchers] want and why they want it. What will [researchers] do with that information, and how will it help my community?” (Participant 20, CAB Focus Group).

Focus group discussions further identified the rationale and strategies to improve recruitment and engagement, including increasing the visibility of research opportunities, offering flexible participation options, and reducing logistical barriers such as transportation and scheduling. One participant described their motivation and ongoing efforts to remain informed about research:

“(I am) trying to empower that way to get people to think more creatively about how they get their care…since my care, I’ve participated as an observer of some of the public forums that they do with the National Institutes of Health.” (Participant 21, CAB Focus Group).

This sentiment was shared by many participants who consider their involvement in research as a duty to ensure that the perspectives of African Americans are included. These findings informed recommendations for future integrative oncology interventions, including culturally responsive programming, group-based formats, and accessible delivery models tailored to the needs of African American individuals with cancer. Their recommendations also highlight the importance of designing research studies that are accessible, responsive, and grounded in community priorities.

## Discussion

This study explored experiences with and attitudes toward integrative oncology (IO) among African American adults with cancer, as well as perspectives on participation in IO research. Participants expressed strong interest in IO for symptom management, emotional support, and overall well-being, alongside persistent barriers to access and concerns about trust and engagement in research.

Participants’ accounts are consistent with a substantial body of literature documenting interpersonal and structural racism across the cancer care continuum. Prior studies have identified racial disparities in patient-provider communication, symptom management, pain treatment, trust in healthcare institutions, and access to supportive care services among African American patients with cancer. Participants in our study described similar experiences, including delayed communication of diagnoses, concerns about differential treatment, and perceptions that longstanding racial stereotypes influenced clinical decision-making. These findings suggest that efforts to expand integrative oncology services must occur alongside broader efforts to address racism and inequity within cancer care delivery.

Within this context, IO emerged as a meaningful resource, offering symptom relief, emotional regulation, and a sense of agency. Participants described using a range of integrative approaches, including acupuncture, spirituality, psychotherapy, and movement-based practices, which align with prior research indicating interest among African American patients in complementary modalities to manage treatment-related side effects and enhance quality of life.

Despite this interest, participants identified barriers to IO access, including limited awareness, scheduling constraints, and cost. While long wait times and limited availability of IO services are widespread, these barriers may be compounded by structural inequities in access to resources, insurance coverage, and health information. Participants also described limited integration of IO into conventional oncology care, often requiring them to seek services independently.

Beyond patient and provider-level factors, participants’ experiences highlight the need for system-level approaches to integrating integrative oncology (IO) into cancer care. While increasing provider awareness and knowledge of IO is important, education alone is unlikely to address the structural barriers identified by participants, including limited access, cost, lack of referrals, and inconsistent availability of services. Implementation research is needed to identify effective strategies for integrating evidence-informed IO services into routine oncology care, particularly in safety-net and community settings that serve historically marginalized populations. Sustainable implementation will require institutional commitment, development of referral pathways, reimbursement mechanisms, workforce training, and care delivery models that support equitable access to whole-person cancer care. These findings suggest that advancing IO for African American cancer survivors will require not only individual behavior change but also healthcare system transformation.

Participants expressed willingness to participate in research, motivated by a desire to improve care and representation. However, this interest was tempered by concerns about trust, transparency, and the perceived extractive nature of research. Participants emphasized the importance of communication, community engagement, and returning findings to participants, underscoring the need for inclusive, accountable research approaches.

The use of individual interviews and focus groups allowed for both in-depth exploration and collective discussion of shared experiences. However, including some participants in both interviews and focus groups may have increased the analytic visibility of certain perspectives.

This study has several limitations. The sample was relatively small and geographically limited to the San Francisco Bay Area, which may affect transferability to other regions and healthcare settings. Participants were English-speaking and had solid malignancies, which may not reflect the experiences of individuals with hematologic cancers or limited English proficiency. These aspects of the study reflect both the clinical populations available at participating sites and the differing treatment trajectories between solid and hematologic cancers. Patients with hematologic malignancies often undergo more intensive, prolonged, and inpatient-focused care,^56-58^ which may shape access to and interest in integrative oncology differently, representing an important area for future research. We also note that participants may have had greater prior exposure to or interest in integrative oncology than the broader cancer population, which may limit transferability to IO-naïve individuals. As a result, the attitudes, preferences, and barriers identified in this study may not fully capture the experiences of individuals with hematologic cancers, highlighting an important area for future research.

Additionally, participants who enrolled may have had a greater interest in integrative oncology, introducing potential selection bias. The inclusion of both patient and Community Advisory Board focus groups also introduced complementary but distinct perspectives.

Despite these limitations, this study contributes to the growing literature on integrative oncology and health equity by centering the perspectives of African American adults with cancer. Improving access to IO may require expanded services, greater integration into oncology care, increased provider awareness, and attention to affordability. Efforts to increase participation in IO research should prioritize transparency, community engagement, and meaningful dissemination of findings.

## Future Directions

Findings from this study suggest several directions for future research and clinical practice to improve access to and delivery of integrative oncology (IO) for Black individuals with cancer. Participants expressed strong interest in IO modalities alongside concerns related to access, communication, and trust, highlighting opportunities to design more responsive and culturally relevant interventions.

Future research may explore interest in specific IO modalities among Black individuals with cancer, including acupuncture, meditation, spirituality-based practices, and yoga. Participants in this study identified these approaches as meaningful components of their healing journeys, suggesting that future intervention studies tailored to these modalities may be both feasible and acceptable. Additionally, future research may evaluate the impact of hybrid delivery models that combine in-person and virtual programming, which may improve accessibility and accommodate participants’ scheduling, transportation, and treatment-related needs.

Feasibility and validation studies examining the use of specific IO modalities in diverse populations are also warranted. Such research may inform the development of culturally responsive interventions and guide the design of larger trials evaluating the impact of integrative approaches on symptom management, quality of life, and cancer survivorship among African American patients.

Participants’ discussions also suggest the importance of relational and culturally concordant care. Future studies may examine the impact of racial concordance between participants and IO facilitators, including African American clinicians and practitioners, as well as the role of peer support and community-building within interventions. These relational components may enhance trust, engagement, and sustained participation.

In addition to research implications, these findings also suggest opportunities for clinical improvement. Participants reported limited communication from providers about available IO services, indicating a need for greater provider education and awareness. Clinical initiatives may focus on informing healthcare providers about patients’ interest in integrative oncology and supporting more proactive communication and referrals.

Participants also emphasized the importance of community awareness and accessibility. Efforts to educate patients and communities about available IO services, along with strategies to reduce logistical barriers, may improve utilization and engagement. Together, these findings support the development of equity-centered integrative oncology programs that are accessible, culturally responsive, and aligned with the priorities of Black individuals with cancer.

## Conclusion

This qualitative study provides insight into African Americans’ interests in and experiences with integrative oncology (IO), as well as their perspectives on participation in research. Our findings highlight the limited availability of integrative health interventions specifically designed for African American individuals with cancer. Although participants recognized the potential benefits of integrative approaches for improving quality of life and managing treatment-related symptoms, these services were not widely accessible due to factors such as limited awareness, logistical barriers, and cost. The qualitative data from this study identified key components of successful integrative health interventions, including addressing cancer treatment–related side effects, providing social support, and ensuring accessibility.

These findings also offer guidance for healthcare professionals seeking to better support Black individuals with cancer. Participants emphasized the need for healthcare providers to offer more information about integrative health options, including physical activity, mind–body practices, and supportive care services. Proactive referrals to integrative health interventions, particularly those that incorporate social support through group-based movement or education-focused programs, may improve engagement and accessibility.

Participants also highlighted the importance of representation and culturally concordant care. Increasing the availability of African American healthcare providers and intervention facilitators may help reduce medical mistrust and enhance participation in integrative oncology. Additionally, because African Americans are often underrepresented in mainstream depictions of integrative health practices, institutions should thoughtfully include diverse representation in promotional materials and program outreach. Such efforts may help Black individuals with cancer feel that integrative health interventions are relevant, accessible, and supportive throughout their cancer journeys.

## Supplemental Material

Supplemental Material - “Doing My Part in my Healing”: A Qualitative Study Exploring Integrative Oncology Practices Among African Americans With Cancer Supplemental Material for “Doing My Part in my Healing”: A Qualitative Study Exploring Integrative Oncology Practices Among African Americans With Cancer by Williams Chanda L, Yang Adrienne, Gilmore Serena, Stanfield Dalila, Paige Steiding, Atreya Chloe and Piawah Sorbarikor in Global Advances in Integrative Medicine and Health.

Supplemental Material - “Doing My Part in my Healing”: A Qualitative Study Exploring Integrative Oncology Practices Among African Americans With Cancer Supplemental Material for “Doing My Part in my Healing”: A Qualitative Study Exploring Integrative Oncology Practices Among African Americans With Cancer by Williams Chanda L, Yang Adrienne, Gilmore Serena, Stanfield Dalila, Paige Steiding, Atreya Chloe and Piawah Sorbarikor in Global Advances in Integrative Medicine and Health.
