# Supplementary material for: “Doing My Part in My Healing”: A Qualitative Study Exploring Integrative Oncology Practices Among African Americans with Cancer
Source: Glob Adv Integr Med Health. 2026 Jul 22;15:27536130261468343. doi: 10.1177/27536130261468343 (PMC13392338; doi:10.1177/27536130261468343)
Supplement: Supplemental Material - “Doing My Part in my Healing”: A Qualitative Study Exploring Integrative Oncology Practices Among African Americans With Cancer [file sj-pdf-1-gam-10.1177_27536130261468343.pdf]

Supplemental Materials Titles and Descriptions --
"Doing my part in my healing": Exploring Integrative Oncology Practices Among African Americans with Cancer

TITLE
Table 1. Demographics
DESCRIPTION
Table 1 presents demographic and clinical characteristics of study participants, including age, gender, cancer type, time since diagnosis, and other relevant participant characteristics. These data describe the sample of African American adults with cancer who participated in interviews and focus groups.

TITLE
Figure 1. Focus Group Image Sort
DESCRIPTION
Figure 1 shows the image-sorting exercise used during the patient focus group to explore participants' perceptions of integrative oncology. Participants were asked to select images that represented how they view integrative oncology and to explain the meaning of each image and the emotions linked to their choices. This activity helped facilitate discussion of participants' ideas about integrative oncology and their personal experiences with integrative and complementary approaches.

TITLE
Table 2. Use of Integrative Medicine
DESCRIPTION
Table 2 summarizes participants' reported use of integrative and complementary approaches during their cancer journey. Modalities include mind–body practices, lifestyle approaches, natural products, and culturally rooted healing practices. The table shows the variety and frequency of integrative modalities used by participants.
