# Supplementary material for: “Doing My Part in My Healing”: A Qualitative Study Exploring Integrative Oncology Practices Among African Americans with Cancer
Source: Glob Adv Integr Med Health. 2026 Jul 22;15:27536130261468343. doi: 10.1177/27536130261468343 (PMC13392338; doi:10.1177/27536130261468343)
Supplement: Supplemental Material - “Doing My Part in my Healing”: A Qualitative Study Exploring Integrative Oncology Practices Among African Americans With Cancer [file sj-pdf-2-gam-10.1177_27536130261468343.pdf]

Appendix A.

Standards for Reporting Qualitative Research (SRQR)

O'Brien B.C., Harris, I.B., Beckman, T.J., Reed, D.A., & Cook, D.A. (2014). Standards for reporting qualitative research: a synthesis of recommendations. *Academic Medicine, 89(9)*, 1245-1251.

No.	Topic	Page No.
**Title and abstract**		
S1	Title	Page 1
S2	Abstract	Page 3
**Introduction**		
S3	Problem formulation	Page 4-5
S4	Purpose or research question	Page 5
**Methods**		
S5	Qualitative approach and research paradigm	Page 5
S6	Researcher characteristics and reflexivity	Page 5
S7	Context	Page 5
S8	Sampling strategy	Page 5
S9	Ethical issues pertaining to human subjects	Page 4
S10	Data collection methods	Page 6
S11	Data collection instruments and technologies	Page 6
S12	Units of study	Page 6
S13	Data processing	Page 6-7
S14	Data analysis	Page 6-7
S15	Techniques to enhance trustworthiness	Page 6-7
**Results/Findings**		
S16	Synthesis and interpretation	Page 7-13
S17	Links to empirical data	Page 7-13
**Discussion**		
S18	Integration with prior work, implications, transferability, and contribution(s) to the field	Page 13-14
S19	Limitations	Page 14
**Other**		
S20	Conflicts of interest	Page 16
S21	Funding	Page 16

[a]The rationale should briefly discuss the justification for choosing that theory, approach, method, or technique rather than other options available, the assumptions and limitations implicit in those choices, and how those choices influence study conclusions and transferability. As appropriate, the rationale for several items might be discussed together.
